# Fine-tuning of a generative neural network for designing multi-target compounds

**DOI:** 10.1007/s10822-021-00392-8

**Published:** 2021-05-28

**Authors:** Thomas Blaschke, Jürgen Bajorath

**Affiliations:** Department of Life Science Informatics and Data Science, B-IT, LIMES Program Unit Chemical Biology and Medicinal Chemistry, Rheinische Friedrich-Wilhelms-Universität, Friedrich-Hirzebruch-Allee 6, 53115 Bonn, Germany

**Keywords:** Multi-target activity, Deep learning, Generative modeling, Structure-promiscuity relationships, Multi-target ligand design

## Abstract

Exploring the origin of multi-target activity of small molecules and designing new multi-target compounds are highly topical issues in pharmaceutical research. We have investigated the ability of a generative neural network to create multi-target compounds. Data sets of experimentally confirmed multi-target, single-target, and consistently inactive compounds were extracted from public screening data considering positive and negative assay results. These data sets were used to fine-tune the REINVENT generative model via transfer learning to systematically recognize multi-target compounds, distinguish them from single-target or inactive compounds, and construct new multi-target compounds. During fine-tuning, the model showed a clear tendency to increasingly generate multi-target compounds and structural analogs. Our findings indicate that generative models can be adopted for de novo multi-target compound design.

## Introduction

Computational de novo and multi-target ligand design are important topics in the pharmaceutical research community. During the early stages of drug discovery, computational compound design is often applied to complement experimental or virtual screening and identify new molecules with desired properties in a time-efficient manner [1]. Recently, deep generative models have become popular for de novo compound design [2, 3]. De novo design using generative models typically involves a two-step process. First, a generative model is trained on a large data set of known compounds using their SMILES [4] representations; then, the model is fine-tuned to generate only compounds with desired properties. The first training step enables the generative model to learn the syntax of molecular string representations and generate new syntactically correct strings without restrictions. For example, Arús-Pous et al. [5] have shown that generative models trained with one million SMILES were capable of covering the chemical space of a fully enumerated set of all possible molecules with up to 13 atoms. Fine-tuning of the generative model is either carried out using reinforcement or transfer learning [6, 7]. In reinforcement learning, the generative model first constructs molecules and then receives property-based feedback for the compounds, for example, by applying a bioactivity classifier. Depending on the feedback, the generative model updates its output to increase or decrease the number of structurally related compounds. Through iterative feedback, the model generates compounds that increasingly meet desired properties. In transfer learning, the model does not rely on feedback when generating compounds, but enters a second learning phase on a smaller subset of compounds with desired properties. By repeatedly exposing the generative model to preferred molecules, it learns common features and then generates new compounds with such features. Both fine-tuning approaches have been applied in different studies to optimize compounds with activity against individual targets [6–11].

Multi-target activity of small molecules, often also referred to as promiscuity, has gained much attention in the medicinal chemistry community over the last two decades. Of note, multi-target activity is often viewed controversially. On the one hand, promiscuity is associated with non-specific ligand-target interactions and assay artifacts caused by aggregators or other assay interference compounds [12–15]. On the other hand, true multi-target activity provides the fundamental basis for polypharmacology of drugs, which is caused by concomitant in vivo interactions with multiple targets [16–19]. Polypharmacology is often essential for therapeutic efficacy in the treatment of multifactorial diseases [16–21], but may also cause undesired side effects. Accordingly, the study of multi-target activity is important not only to better understand the fundamental basis of polypharmacology, but also to control potential side effects of new drugs. To rationalize multi-target activity and predict multi-target compounds, different computational approaches have been adopted [17, 22, 23]. Most of these predictions have focused on the identification of additional targets for known active compounds [24–31], while only few have attempted to predict different types of promiscuous compounds directly [32–35]. These latter studies have shown that promiscuous and non-promiscuous compounds could be differentiated with reasonable accuracy on the basis of chemical structure, indicating the presence of structural patterns that distinguish compounds with single- and multi-target activity. These studies have also revealed that nearest neighbor relationships between multi-target or single-target compounds strongly contributed to the predictions. Further exploring structure-promiscuity relationships is expected to aid in the design of compounds with pre-defined multi-target activities, which is currently mostly attempted by combining pharmacophore information for different targets [36–39]. However, another potential route to designing multi-target compounds would be adapting deep generative models for this task, which to our knowledge has not been attempted so far.

Herein, we explore the possibility of fine-tuning a SMILES-based generative neural network to recognize multi-target compounds, distinguish them from single-target or inactive compounds, and construct new multi-target candidates. If structural patterns exist that are characteristic of multi-target compounds, a generative model should be able to detect these patterns via transfer learning and utilize them to create new multi-target compounds. In contrast to other machine learning algorithms, SMILES-based generative models do not rely on the explicit calculation of substructure fingerprints or physiochemical properties. Neither use these models the information that compounds have multi-target activity, nor are they specifically trained to distinguish between multi- and single-target compounds. This added layer of abstraction enables the recognition of non-obvious structure-promiscuity relationships in an unsupervised manner, thereby bridging between transfer learning and multi-target ligand design.

For this study, we used high-confidence compound sets extracted from biological screening data and applied the publicly available REINVENT model [40] for transfer learning. Through fine-tuning, we determined if the generative model was able to recognize multi-target compounds and distinguish them from single-target or inactive compounds. Furthermore, newly generated SMILES representations were analyzed following each fine-tuning cycle to assess the recovery of known compounds and structural neighbors as a proof-of-concept measure for the principal capacity of the model to generate new multi-target compounds.

## Methods and materials

### Data extraction

For the analysis, a comprehensive collection of publicly available PubChem screening data [41] was used after applying a number of confidence criteria. Only qualitative compound assay results for human targets with the designation ‘active’ and ‘inactive’ were considered. Assays imported from ChEMBL [42], BindingDB [43], or Tox21 [44] and revoked or ambiguously annotated assays were excluded from the analysis. Because the assessment of multi-target compounds is particularly vulnerable to false positive activity assignments, assays with a hit rate higher than 2% were also excluded. Furthermore, compounds with potential liabilities were omitted including designated pan-assay interference compounds [45] detected with publicly available filters from ChEMBL, ZINC [46], and RDKit [47], molecules violating empirical medicinal chemistry rules [48], and others yielding aggregation alerts [49]. Finally, compounds with inconsistent activity annotations across different assays for the same target were also discarded.

Qualifying compounds were assigned to three different sets depending on the number of human targets they were active against. Screening molecules with activity against five or more different targets were classified as multi-target compounds. In addition, compounds with activity against only one target and confirmed inactivity against at least four other targets were categorized as single-target compounds. Furthermore, compounds with no reported activity but inactivity in assays against at least five different targets were classified as inactive (“no-target”) compounds. Qualifying compounds not meeting any of these selection criteria were not further considered. Multi-target compounds were required to be active against at least five different targets to ensure that they were promiscuous in nature, setting them clearly apart from single-target compounds.

### Generative model

For generative modeling, we used REINVENT, a publicly available model that was originally trained on ~ 1.4 million bioactive compounds from ChEMBL [40].

For our study, REINVENT was fine-tuned using a random selection of 1000 multi-target compounds. Fine-tuning was carried out for 200 epochs using the ADAM optimizer [50]. The loss function used during fine-tuning minimized the negative log-likelihood (NLL) of the SMILES of multi-target training compounds. After each training epoch, the NLL for the canonical SMILES of all detected compounds was calculated. To avoid overfitting, the SMILES representations of multi-target compounds were randomized [51].

### Compound design

After each fine-tuning epoch, 1,000,000 SMILES were sampled. The sampling of molecules over different epochs was performed using the same random seed. Accordingly, two identical models would sample the same SMILES. Consequently, any difference in the sampled SMILES directly resulted from fine-tuning of the underlying generative model and was not a result of random sampling. The generated SMILES were canonicalized with RDKit and all unique valid SMILES were considered to represent newly generated compounds.

### Molecular similarity

For each generated molecule, the extended-connectivity fingerprint with bond diameter 6 (ECFP6) [52] and constant 2048 bit format was used as a representation and Tanimoto similarity to multi-, single-, and no-target compounds from PubChem was calculated. If the Tanimoto similarity of a generated molecule to a PubChem compound was at least 0.6, it was classified as a structural (fingerprint) neighbor.

## Results and discussion

### Compound sets

Applying high-confidence data selection criteria taking positive as well as negative assay results into account, 2809 multi-target, 61,928 single-target, and 295,395 no-target compounds were extracted from PubChem screening assays. As expected, the compound data set was imbalanced, containing comparably few multi-target compounds.

A random selection of 1000 multi-target compounds was used as a training set for fine-tuning the general-purpose REINVENT model. The remaining 1809 multi- and 61,928 single-target compounds, as well as an equally sized random subset of 61,928 no-target compounds, were used as test sets.

### Fine-tuning

**Fig. 1:**
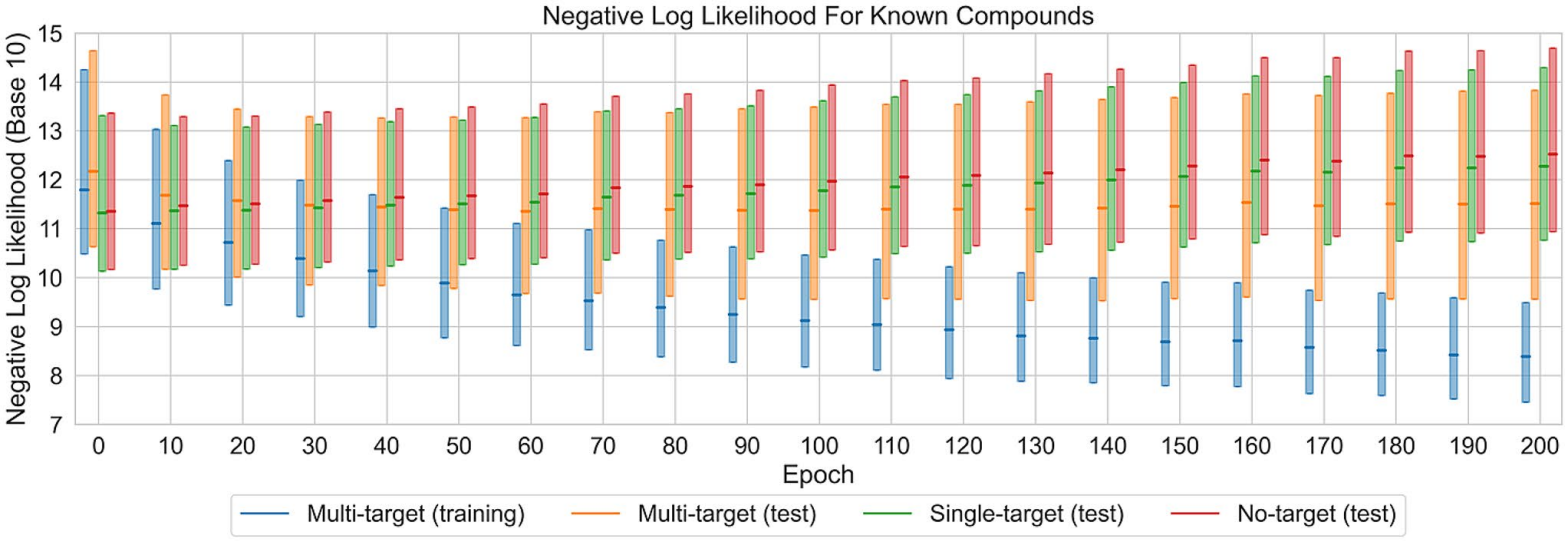
Distribution of negative log-likelihood values. Boxplots report the NLL distribution for known multi-, single-, and no-target compounds. Shown are the 25% quartile, the median (horizontal line), and the 75% quartile. Whiskers and statistical outliers are omitted for clarity

The REINVENT model was fine-tuned for 200 epochs. After each epoch, the NLL for all PubChem compounds was calculated. NLL values provide a quantitative estimate for the probability that the model will (re-)generate a particular compound at a given stage in the process. The resulting NLL value distribution is shown in Fig. 1.

Prior to fine-tuning (epoch 0), there was a notable difference in the NLL distribution between multi-, single-, and no-target compounds. On the basis of the NLL median values, multi-target compounds were 3–4 times less likely to be generated than single- or no-target compounds. Moreover, comparing the 75% quartile, multi-target compounds were 10 times less likely than single- or no-target compounds.

This difference in the likelihood of generating multi-target compounds compared to others could be rationalized by considering the derivation of the generative REINVENT model [40]. The REINVENT model was originally trained on a large collection of bioactive compounds from ChEMBL, the majority of which are single-target compounds [53]. ChEMBL only contains a very small proportion of compounds with reported activity against more than five targets (< 2%), which has remained essentially constant over time [53]. Accordingly, the REINVENT model was tailored towards single-target compounds. Prior to fine-tuning, the model also preferentially learned structural features from single-target screening compounds (but also inactive compounds), as revealed by the higher likelihood of generating single- and no-target compounds from screening assays; an interesting observation.

However, after only 10 epochs of fine-tuning, the NLL value distributions for multi-, single-, and no-target compounds were very similar including their mean values. At this stage, the 25% and 75% quartile displayed a difference of less than 0.4 NLL units.

After 30 epochs of fine-tuning, the model started to preferentially recognize multi-target compounds from the training set. Compared to the initial state prior to fine-tuning, the median NLL was reduced by 1.5 units. This reduction already corresponded to a 400-fold increase in the likelihood to generate multi-target compounds at this early stage. Moreover, for the 75% quartile, the median NLL was reduced by 2.2. Concomitantly, the NLLs for single- and no-target compounds slightly increased. After 50 epochs, the median NLL value for multi-target test compounds was lowered relative to the median for the other training compounds and the difference further increased during fine-tuning. Similarly, the median NLL for multi-target training compounds consistently decreased during fine-tuning, as monitored in Fig. 1. After 200 epochs, the median NLL approached a value of 8. Taken together, these observations indicated that the model increasingly learned structural features shared by multi-target training and test compounds and discriminated single- and no-target compounds, consistent with the underlying design idea.

### Generating multi-target compounds

**Fig. 2:**
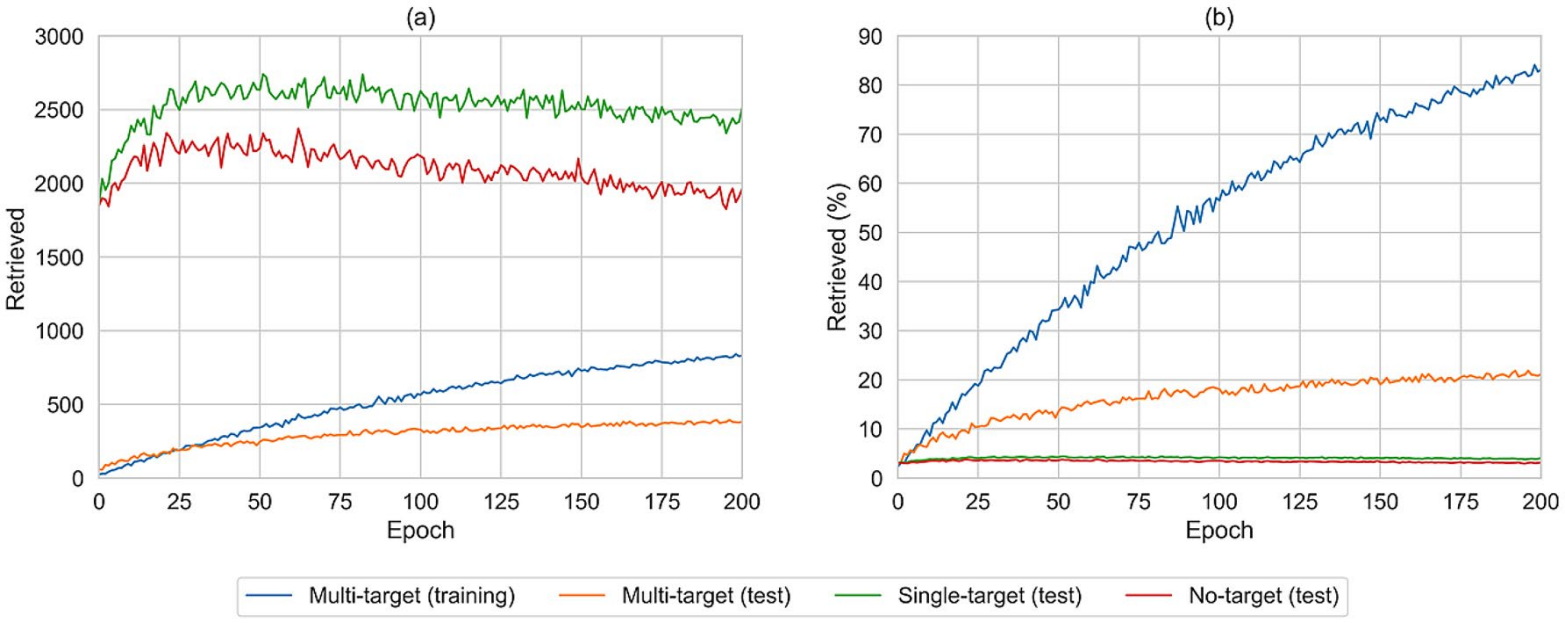
Compound retrieval during fine-tuning. Reported is the number of retrieved multi-, single-, and no-target compounds among 1,000,000 SMILES sampled after each fine-tuning epoch. **a** shows the absolute number of retrieved compounds and **b** the percentage for each data set

Of note, the NLL values were exclusively calculated for the canonical SMILES of each compound. Since a compound may also be represented by a variety of non-canonical SMILES strings, the likelihood of generating a compound is expected to be underestimated by the NLL calculated on the basis of its canonical SMILES representation. Therefore, to more comprehensively monitor the ability of the generative model to create multi-target compounds, 1,000,000 SMILES were sampled randomly after each epoch of fine-tuning, canonicalized, and filtered for PubChem compounds. The results are shown in Fig. 2.

Prior to fine-tuning, the model generated 3% of known compounds across the three different sets, with no significant difference between multi-, single-, and no-target compounds. After 25 epochs of fine-tuning, 20% of the multi-target training set, 10% of the multi-target test set, and 4% of both the single-target and no-target test sets were retrieved. Throughout fine-tuning, the number of reproduced multi-target training and test compounds increased. After 200 epochs, 85% of the multi-target training and 21% of the test set were reproduced, in contrast to only 4% of the single-target and 3% of the no-target test set. The increase in the number of multi-target test set compounds provided firm evidence that the model recognized structure-promiscuity patterns in the training set and used these patterns to preferentially generate multi-target compounds.

### Neighbors of multi-target compounds

**Fig. 3:**
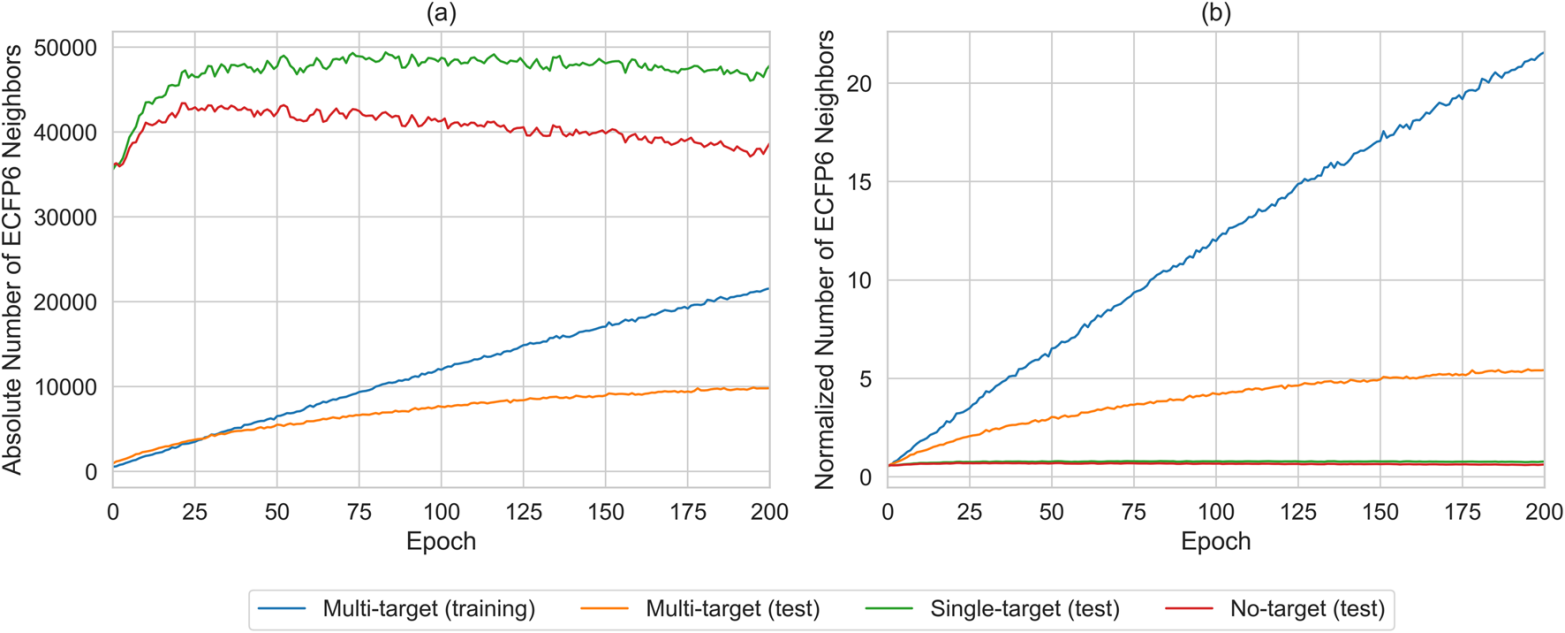
Distribution of structural neighbors. Reported is the number of generated (fingerprint) neighbors of multi-, single-, and no-target compounds among 1,000,000 SMILES sampled after each fine-tuning epoch. Duplicated canonical SMILES were removed and only unique, valid, and novel SMILES were considered. **a** shows the absolute number of generated neighbors and **b** the normalized number of generated neighbors per known compound

Nearest neighbor relationships were previously found to play an important role in distinguishing between different types of promiscuous and non-promiscuous compounds using supervised machine learning [33–35]. The influence of nearest neighbor relationships indicated that promiscuous compounds were typically more similar to other promiscuous than non-promiscuous compounds and vice versa [35]. To analyze structural neighbors of all generated compounds, Tanimoto similarity to other training and test compounds was calculated (excluding exactly reproduced compounds). As a neighbor criterion, an ECFP6 similarity threshold of 0.6 was applied, thus focusing on closely related compounds. To account for the difference in size between the multi-target test set and the single- and no-target test sets, the number of detected neighbors was normalized relative to the size of each set. The results are shown in Fig. 3 (and essentially parallel the observations made in Fig. 2). Prior to fine-tuning, 3% of the generated compounds were structural neighbors of PubChem training and test compounds. Over the first 30 epochs of fine-tuning, the number of generated neighbors increased for the training and test sets. After epoch 50, the absolute number of generated neighbors for the no-target test set decreased to 38,000 compounds but remained constant at 48,000 compounds for the single-target set. For the multi-target training and test set, the number of neighbors increased throughout fine-tuning to 22,000 and 10,000 compounds, respectively (corresponding to 22 neighbors per training and five neighbors per test compound). The large number of neighbors generated for multi-target compounds provided further evidence for the ability of the fine-tuned model to recognize characteristic structural patterns and create structural analogs.

**Fig. 4:**
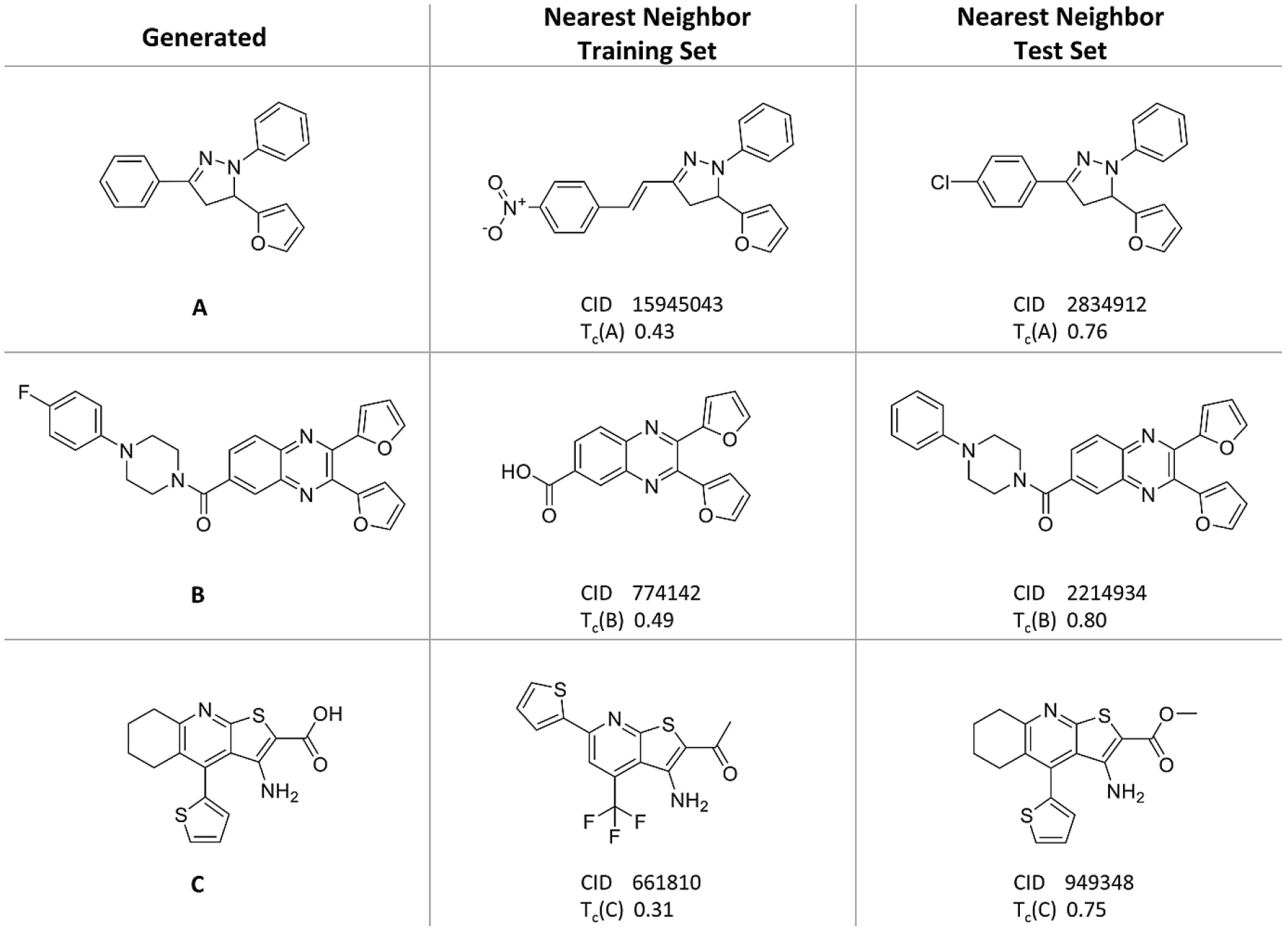
Exemplary compounds. Shown are three examples of newly generated multi-target compounds together with their nearest neighbors from the multi-target training and test set, respectively. In each case, the calculated ECFP6 Tanimoto coefficient (T_c_) is reported

Figure 4 shows examples of newly generated multi-target candidate compounds and their nearest neighbors from the training and test set. In all three instances, structural modifications compared to the nearest neighbor from the training set produced candidate molecules that closely resembled test set compounds, hence illustrating the ability of the fine-tuned model to sample chemical space populated by multi-target compounds. The generation of such analogs complemented the capacity of the model to reproduce known multi-target compounds, which was monitored as a quality criterion.

### Scaffold analysis

**Fig. 5:**
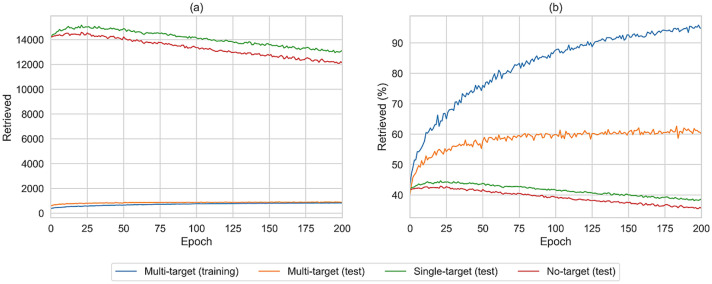
Scaffold retrieval during fine-tuning. Reported is the number of multi-, single-, and no-target BM scaffolds detected in 1,000,000 SMILES sampled after each fine-tuning epoch. **a** shows the total number of BM scaffolds and **b** the percentage for each data set

In addition to nearest neighbor analysis, we also assessed the similarity between the newly generated compounds, training set, and test set compounds on the basis of Bemis and Murcko (BM) scaffold composition [54]. From each compound, the BM scaffold was extracted and scaffolds of newly generated, training, and test set compounds were compared. The multi-target training set was found to contain 869 unique BM scaffolds and the multi-target test set 1463 BM scaffolds, 1252 of which (86%) were not present in the training set. Furthermore, the single-target and no-target test sets yielded 33,977 and 34,024 BM scaffolds, respectively, 335 and 245 of which were present in the multi-target training set, respectively. We then determined scaffolds from each data set that were generated during fine-tuning. The results are shown in Fig. 5. Prior to fine-tuning, the model retrieved ~ 41% of the BM scaffolds from the training and test sets. Over the first 25 epochs of fine-tuning, the total number of retrieved BM scaffolds increased for the training and all test sets. After epoch 25, the number of retrieved scaffolds decreased for the no-target and single-target test set to 12,119 (36%) and 13,052 (38%), respectively. For the multi-target training and test set, the number of retrieved BM scaffolds increased throughout fine-tuning to 833 (96%) and 905 (62%), respectively. Remarkably, the majority of the retrieved BM scaffolds for the multi-target test set (698 of 905; 77%) were not present in the training set. Hence, scaffold analysis provided further evidence for the ability of the fine-tuned model to recognize structural characteristics of multi-target compounds.

### Compound classification

To further explore newly generated compounds, we trained a decision tree ensemble classifier using the gradient boost algorithm from the XGBoost library [55]. The classifier was built to distinguish multi-target compounds from single- and no-target compounds. It was derived using the multi-target training set (positive class label) and combined random subsets of 30,000 single- and no-target compounds each (negative class label). Using the remaining screening compounds as a test set, the classifier reached a ROC AUC score of 0.82, a Matthews correlation coefficient (MCC) of 0.30, and recall of 0.32, hence confirming reasonable accuracy.

Applying the classifier, prior to fine-tuning, 2.4% of the generated compounds were labeled as multi-target compounds. During fine-tuning, the fraction of compounds classified as multi-target compounds steadily increased. After 200 epochs, 26.6% of the newly generated compounds were predicted to be multi-target compounds. Thus, compound classification also supported the ability of the fine-tuned model to preferentially generate multi-target compounds.

## Conclusion

In this work, we have attempted to fine-tune a deep generative model originally trained on bioactive compounds for de novo design for recognizing and producing multi-target compounds. Therefore, high-confidence data sets of multi-, single-, and no-target (inactive) screening compounds were assembled considering positive and negative assay results. Using a subset of known multi-target compounds, the publicly available REINVENT model was fine-tuned using transfer learning, and its ability to re-generate known multi-, single-, and no-target compounds was evaluated on the basis of NLL analysis. Consistent with its derivation, the original REINVENT model was tailored towards the generation of single-target compounds, but also recognized no-target compounds. However, fine-tuning via unsupervised transfer learning systematically increased the likelihood of generating multi-target compounds, while decreasing the likelihood of producing single- or no-target compounds. During fine-tuning, the model regenerated known multi-target test compounds at increasing rates, in contrast to single- or no-target compounds. Moreover, the analysis of structural neighbors of training and test compounds, scaffold assessment, and compound classification studies further supported the ability of the fine-tuned model to particularly generate multi-target candidate compounds. Taken together, the results also provided evidence for the presence of structure-promiscuity relationships that were detected, learned, and utilized by the model, consistent with earlier findings. Notably, corresponding structural patterns were captured by randomized SMILES of multi-target compounds used for the fine-tuning and recognized in an unsupervised manner. Taken together, our findings provide proof-of-concept for generative de novo multi-target compound design. As a part of our study, the data sets and custom code generated for our analysis have been made freely available [56].
